# cAMP response element–binding protein: A credible cancer drug target

**DOI:** 10.1016/j.jpet.2025.103529

**Published:** 2025-03-04

**Authors:** Jinghui Hong, Yuheng Wu, Mengxin Li, Ki-Fong Man, Dong Song, Siang-Boon Koh

**Affiliations:** 1 Department of Breast Surgery, General Surgery Centre, The First Hospital of Jilin University, Changchun, Jilin, China; 2 Faculty of Health and Life Sciences, University of Bristol, Bristol, United Kingdom; 3 University Hospitals Bristol and Weston, National Health Service (NHS) Foundation Trust, Bristol, United Kingdom

**Keywords:** cAMP response element binding protein (CREB), Transcription factor, Anticancer drug, Therapy resistance, Biomarker, Breast cancer

## Abstract

Despite advancements in radiotherapy, chemotherapy, endocrine therapy, targeted therapy, and immunotherapy, resistance to therapy remains a pervasive challenge in oncology, in part owing to tumor heterogeneity. Identifying new therapeutic targets is key to addressing this challenge because it can both diversify and enhance existing treatment options, particularly through combination regimens. The cAMP response element–binding protein (CREB) is a transcription factor involved in various biological processes. It is aberrantly activated in several aggressive cancer types, including breast cancer. Clinically, high CREB expression is associated with increased breast tumor aggressiveness and poor prognosis. Functionally, CREB promotes breast cancer cell proliferation, survival, invasion, metastasis, as well as therapy resistance by deregulating genes related to apoptosis, cell cycle, and metabolism. Targeting CREB with small molecule inhibitors has demonstrated promise in preclinical studies. This review summarizes the current understanding of CREB mechanisms and their potential as a therapeutic target.

**Significance Statement:**

cAMP response element–binding protein (CREB) is a master regulator of multiple biological processes, including neurodevelopment, metabolic regulation, and immune response. CREB is a putative proto-oncogene in breast cancer that regulates the cell cycle, apoptosis, and cellular migration. Preclinical development of CREB-targeting small molecules is underway.

## 1 Introduction

The cAMP response element–binding protein (CREB) is a transcription factor that plays a pivotal role in various biological processes (Shaywitz and Greenberg, 1999). CREB is encoded by the *CREB1* gene (Dinevska et al, 2024). Aberrant activation of CREB has been found to be closely associated with the onset and progression of multiple cancer types. Specifically, numerous studies have demonstrated the role of CREB in promoting tumor cell proliferation (Shankar and Sakamoto, 2004), apoptotic evasion (Mayr and Montminy, 2001), invasion (Kang et al, 2022), and metastasis (Wu et al, 2007), collectively suggesting that CREB augments tumor growth and expansion.

Breast cancer is one of the most prevalent malignancies globally. According to the Global Cancer Statistics 2022, approximately 2.3 million new breast cancer cases were diagnosed worldwide, resulting in an estimated 665,000 deaths (Bray et al, 2024). Besides surgery, the primary treatment modalities for this disease are radiotherapy, chemotherapy, endocrine therapy, targeted therapy, and immunotherapy (Curigliano et al, 2023). Despite this array of therapeutic options, therapy resistance is a bottleneck in improving curative rates (Barzaman et al, 2020). Therefore, identifying alternative therapeutic targets represents a key toward improving the efficacy of current treatment options. For example, advancement in targeted therapies such as poly (ADP-ribose) polymerase inhibitors and antibody–drug conjugate sacituzumab govitecan has dramatically improved the survival rates and quality of life in a subset of patients with breast cancer (Li et al, 2022).

In the context of breast cancer, CREB has been shown to promote malignancy by modulating genes associated with growth-promoting pathways (Xiao et al, 2010; Zhang et al, 2015; Chelakkot et al, 2023). For instance, CREB upregulates antiapoptotic genes, bolstering cellular resistance to apoptosis while activating genes governing cell cycle regulation and metabolism to fuel cancer cell proliferation (Shankar et al, 2010). Moreover, CREB facilitates breast tumor invasion and metastasis by orchestrating the expression of genes that regulate angiogenesis and cell migration (Mayr and Montminy, 2001; Tsui et al, 2019). Accordingly, high expression levels of CREB have been observed across multiple breast cancer subtypes, with its activity strongly correlating with poor prognosis in patients (Chhabra et al, 2007). Emerging studies using therapeutic interventions that target CREB have demonstrated preclinical efficacy in suppressing breast cancer cell growth, suggesting that CREB is a viable therapeutic target for this disease (Sapio et al, 2020). Previous reviews have reported on the biochemistry and general role of CREB in human pathology (Steven et al, 2020; Zhang et al, 2020; Chowdhury et al, 2023). In this review, we focus on its role in cancer, particularly breast cancer where the strongest evidence is present, and provide an update on the development of small molecules that act on this transcription factor.

## 2 The structure and activation mechanisms of CREB

**Fig. 1:**
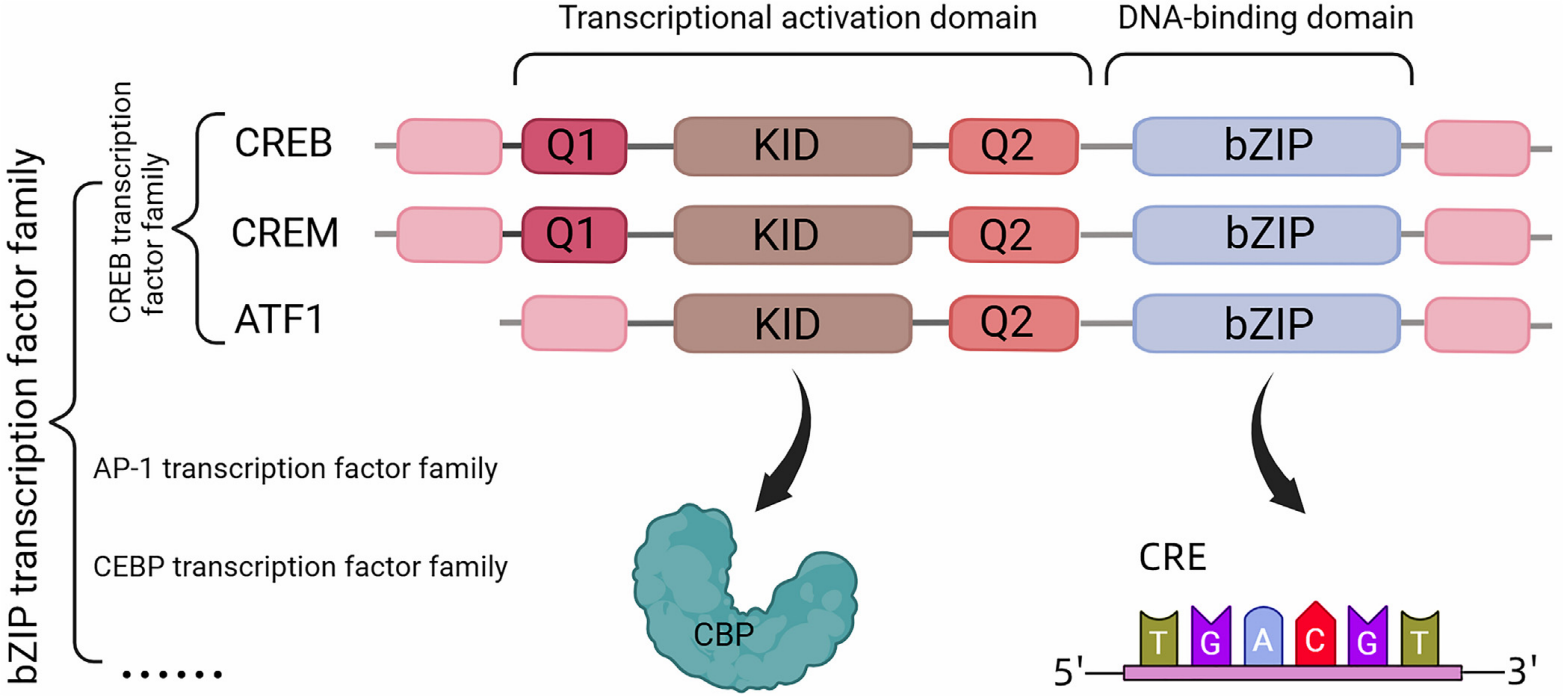
Protein domains of members of the CREB transcription factor family. The CREB family belongs to the bZIP family of transcription factors. The CREB family consists of CREB, CRE modulator (CREM), and activating transcription factor 1 (ATF1), each of which contains functionally important KID and bZIP domains. The KID binds primarily to the KID interacting domain on the CBP, whereas the bZIP domain binds to the CRE in the promoter region of a target gene and initiates gene transcription. AP-1, activator protein 1; CEBP, CCAAT-enhancer-binding protein.

First identified in 1987 as a substrate for phosphorylation, CREB is a member of the basic leucine zipper (bZIP) superfamily, where it harbors a basic region rich in lysines and arginines, as well as a leucine zipper (Mayr and Montminy, 2001; Martin and Nguyen, 2022). It is considered part of the CREB transcription factor family, which includes 2 other structurally and functionally similar proteins, namely cAMP response element (CRE) modulator and activating transcription factor 1 (De Cesare and Sassone-Corsi, 2000; Li et al, 2019) (Fig. 1). These 3 transcription factors share a highly homologous bZIP domain and are regulated by the cAMP signaling pathway (Lalli and Sassone-Corsi, 1994). In addition to homodimerization, members of the CREB transcription factor family can form heterodimers among themselves and thus coregulate the expression of multiple genes (Hai and Curran, 1991; Loriaux et al, 1994). Broadly, these genes are associated with cell proliferation, differentiation, and metabolism (Sapio et al, 2020).

At the protein level, CREB is characterized by 4 key domains: a kinase-inducible domain (KID), a bZIP domain, and 2 glutamine-containing domains (Steven and Seliger, 2016; Shnitkind et al, 2018). The KID, an activation domain, contains the crucial serine 133 site (Ser133) phosphorylation site (Gonzalez and Montminy, 1989). Activation of CREB by the cAMP signaling pathway occurs via phosphorylation at this site, which enhances the physical association between CREB and the CREB-binding protein (CBP). The CREB-CBP complex, which is mediated by the KID interacting domain in CBP, enables the initiation of transcriptional activation (Chrivia et al, 1993; Asahara et al, 2001). The bZIP domain, which includes the basic region and the leucine zipper, functions as the DNA-binding domain (Hoeffler et al, 1988). The basic region recognizes and binds to the CRE sequence on the DNA, while the leucine zipper facilitates the dimerization of CREB, forming either homodimers or heterodimers with other bZIP family members (Yun et al, 1990; Karpinski et al, 1992). Additionally, CREB contains 2 glutamine-rich domains, Q1 and Q2. The Q1 domain, located at the N terminus, serves as a basal transcriptional activation domain. On the other hand, the Q2 domain, located at the C terminus, acts as a strong transcriptional activation domain that recruits and binds to coactivators to initiate gene transcription (Brindle et al, 1993; Johannessen et al, 2004b; Martinez-Yamout et al, 2023). Several known coactivators of CREB include TATA-box–binding protein–associated factor 4 and RNA polymerase II (Johannessen et al, 2004b).

**Fig. 2:**
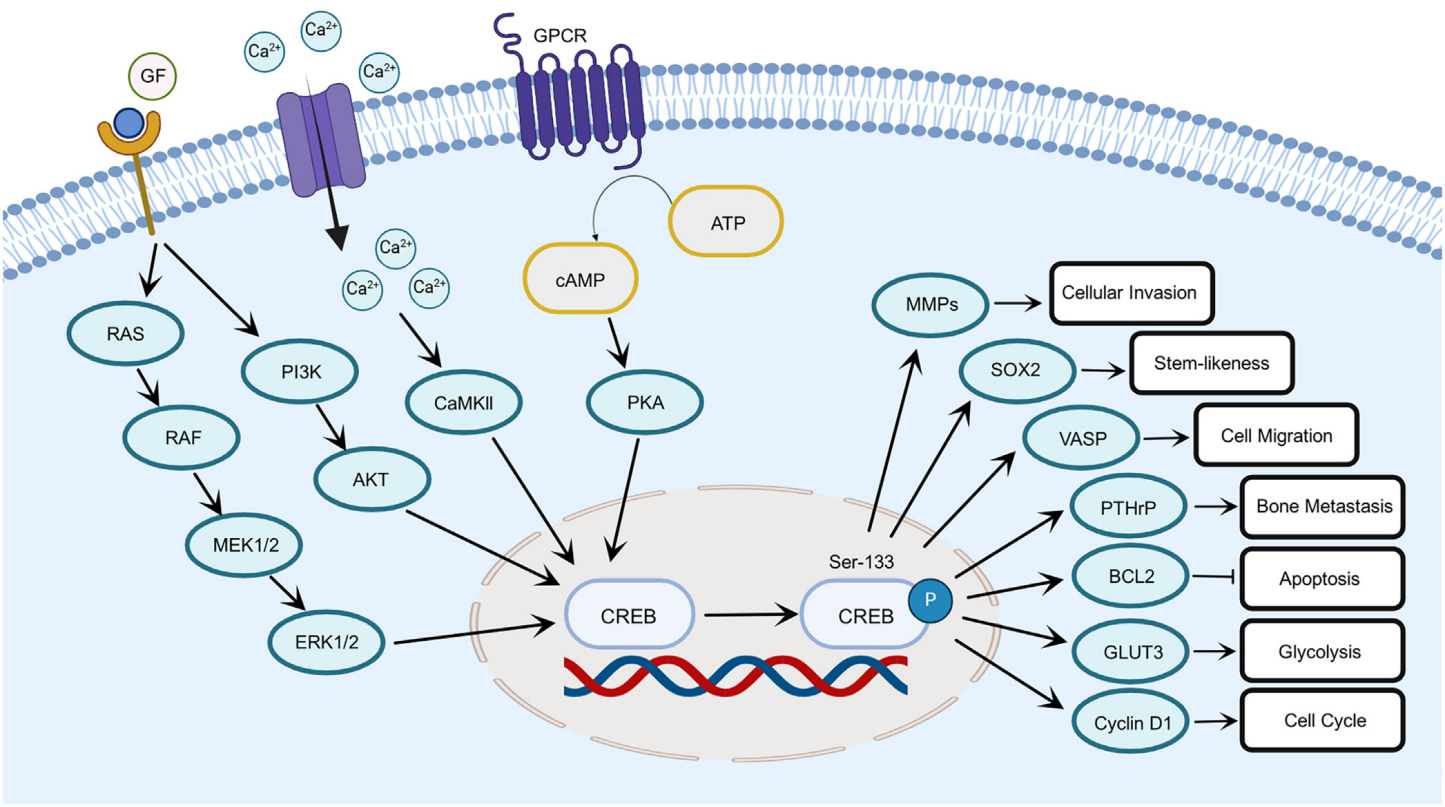
Upstream and downstream signaling associated with CREB. A multitude of factors including growth factors, cytokines, stress, and calcium induce phosphorylation of CREB via the CaMK, MAPK, PI3K, and PKA signaling pathways. Phosphorylated CREB regulates the expression of protumor genes such as BCL2, MMPs, SOX2, and cyclin D1 to drive the biological functions of breast cancer cells. PTHrP, parathyroid hormone–related peptide; VASP, vasodilator-stimulated phosphoprotein.

CREB is activated by a myriad of cellular signaling axes, all converging primarily on its phosphorylation at the Ser133 residue (Shaywitz and Greenberg, 1999). Kinases responsible for this phosphorylation include protein kinase A (PKA), Ca2+/calmodulin-dependent protein kinases (CaMKs), mitogen-activated protein kinases (MAPKs), and phosphoinositide 3-kinases (PI3Ks) (Lonze and Ginty, 2002) (Fig. 2). First, extracellular signals such as neurotransmitters and hormones can activate adenylate cyclase via G protein–coupled receptors, leading to an increase in intracellular cAMP concentration. Elevated cAMP levels activate PKA, which in turn phosphorylates the Ser133 residue of CREB, inducing its activation (Altarejos and Montminy, 2011). Second, increased intracellular calcium levels can activate the CaMKs pathway, similarly promoting CREB phosphorylation (Esvald et al, 2020). Third, cytokines and growth factors can also activate CREB through the MAPK/PI3K pathway, whereby effectors ERK1/2 and AKT phosphorylate CREB at Ser133 (Ahmed et al, 2022). The integration of these signaling pathways thus enables CREB to orchestrate the regulation of multiple biological processes, including cell growth, survival, and neuroplasticity (Saura and Cardinaux, 2017; Esvald et al, 2020; Bal et al, 2023). Once phosphorylated, CREB binds to coactivator CBP or histone acetyltransferase p300, forming a transcriptional activation complex (Johannessen et al, 2004a; Wang et al, 2013). This complex promotes gene transcription by binding to the CRE sequence on the DNA, thereby initiating the expression of target genes (Kilanowska et al, 2022).

## 3 The pathophysiologic functions of CREB

CREB has critical functions in neurodevelopment, metabolic regulation, and immune response; thus, its deregulation can considerably alter normal cell biology (Revilla and Granja, 2009; Han et al, 2020; Zhang et al, 2022a). In neuroplasticity, CREB is implicated in promoting neuronal growth, survival, and synaptic plasticity by regulating the expression of nerve growth factor as well as brain-derived neurotrophic factor (Barco and Marie, 2011; Belgacem and Borodinsky, 2017). It is also a key regulator of long-term potentiation and long-term depression, which are essential mechanisms underpinning learning and memory (Sakamoto et al, 2011). For example, CREB Ser133A mutant mice were found to exhibit impaired long-term potentiation and spatial cognition, demonstrating the importance of CREB in mammalian cognition and memory regulation (More et al, 2022). Another study involving Purkinje neurons from mouse cerebellar cultures transfected with the CREB gene demonstrated that CREB is involved in establishing long-term depression (Ahn et al, 1999). In metabolic regulation, CREB is involved in processes such as glucose and lipid metabolism (Herzig et al, 2003; Dentin et al, 2008). It controls lipid metabolism by regulating gluconeogenesis through the nuclear receptor coactivator peroxisome proliferator-activated receptor gamma coactivator-1 (Herzig et al, 2001). Additionally, CREB promotes insulin resistance by upregulating the expression of transcriptional repressor activating transcription factor 3, which in turn downregulates the expression of insulin-sensitive glucose transporter protein 4 (Qi et al, 2009). In the immune system, CREB is a vital regulator whereby it can inhibit nuclear factor-*κ*B (NF-*κ*B) activation in immune cells, as well as promote the proliferation of T and B lymphocytes (Wen et al, 2010). In one study, the authors found that the carboxyl-terminal region (amino acids 286-551) of NF-*κ*B p65 and phosphorylated CREB competitively interact with the same region of CBP, thus dictating the level of NF-*κ*B activation (Parry and Mackman, 1997). Notably, CRE sequences have been found in the promoter region of numerous T cell-specific genes (eg, TCR*α*, CD8*α*, and IL-2), underlining the regulatory role of CREB in these genes (Wen et al, 2010). Additionally, as an antiapoptotic transcription factor in macrophages, CREB is crucial in regulating innate immunity (Park et al, 2005). For example, CREB is a p38 MAPK-regulated transcription factor required for macrophage survival. Specifically, p38 activates CREB to induce the transcription of antiapoptotic genes, plasminogen activator inhibitor-2, and Bfl-1/A1 (Park et al, 2005).

When aberrantly expressed, CREB plays a protumor role through multiple mechanisms (Steven et al, 2020). It is involved in the transcription of proliferation and survival genes such as cyclin D1 and BCL-2, promoting cell cycle progression and inhibiting apoptosis (Desdouets et al, 1995; Persengiev and Green, 2003; Yan et al, 2013). In addition, CREB facilitates tumor angiogenesis by modulating the expression of vascular endothelial growth factor, enabling the supply of oxygen and nutrients to the tumor niche (Wu et al, 2007; Hu et al, 2016). Furthermore, CREB promotes tumor cell invasion and metastasis by controlling the expression of genes such as matrix metalloproteinases (MMPs) and E-cadherin, which in turn affect intercellular adhesion and matrix degradation (Nyormoi and Bar-Eli, 2003). CREB also plays a role in the tumor microenvironment (TME) by directly influencing the activity of tumor-associated immune cells, thereby aiding tumor cells in evading immune surveillance (Dinevska et al, 2024). For example, in colorectal cancer cells, CREB binds to the promoter of programmed death ligand-1 and directly activates programmed death ligand-1 expression, promoting immune escape (Li et al, 2023). Finally, CREB is implicated in the development of drug resistance in tumor cells through its regulation of drug transporter protein expression. This modulation influences drug concentration and metabolism within the cell, thus impacting the sensitivity of tumor cells to chemotherapeutic agents. For example, CREB activation in colorectal cancer cells suppresses the expression of P-glycoprotein, a membrane transporter protein (also called drug efflux pump) associated with drug resistance encoded by the multidrug resistance 1 gene (Wang et al, 2015). In another study using prostate cancer as the experimental model, it has been found that upregulation of ATP-binding cassette subfamily G member 4 induced by the NF-*κ*B-c-MYC-CREB axis drives the development of chemoresistance (Mallappa et al, 2019). Similarly, it has been found that RTK-like orphan receptor 2 expression in prostate cancer significantly increased following pharmacologic inhibition of the androgen receptor pathway. This increase in turn upregulates the expression of the lineage commitment transcription factor ASCL1 via the ERK/CREB signaling pathway, thereby promoting tumor progression and androgen receptor pathway inhibitor resistance (Tabrizian et al, 2023).

## 4 The role of CREB in breast cancer

Numerous studies have demonstrated a significant elevation of CREB expression in breast cancer tissues compared with normal tissues (Chhabra et al, 2007; Fan et al, 2012; Xin et al, 2020). Accordingly, CREB is commonly perceived to function as a proto-oncogene in this disease (Fig. 2). Upstream, several factors regulate the activity of CREB, including growth factors, cytokines, hormones, stress, microRNAs (miRNAs), and noncoding RNAs (Steven et al, 2020). Among these regulators, in the context of breast cancer, a few recent studies have demonstrated the roles of miRNA-450a and long noncoding RNA TDRKH-AS1 in regulating CREB (Zhang et al, 2022b; Ding et al, 2023). However, the possibility of a feedback loop between CREB and miRNAs, which could in turn affect the expression and function of miRNAs, remains untested (Wang et al, 2016b). Investigating this potential feedback loop could unveil targetable nodes along this regulatory axis of CREB in breast cancer.

Current studies on CREB have established its role in promoting breast cancer cell proliferation, inhibiting apoptosis, enhancing invasion and metastasis, and creating a tumor-permissive microenvironment. Similar to its mechanism of action in promoting the growth of other tumor types, CREB enhances the proliferation of breast cancer cells by regulating the transcriptional activity of multiple target genes. This activation leads to the expression of cell cycle proteins (eg, cyclin D1) and proliferation-related factors (eg, EGR1), thereby accelerating the cell cycle (Boulon et al, 2002; Shankar and Sakamoto, 2004; Hu et al, 2019). In terms of apoptosis, the positive correlation between CREB and the antiapoptotic factor BCL-2 has been validated in a number of independent breast cancer studies (Shankar et al, 2010; Zhou et al, 2015; Liang et al, 2023). For example, we have recently found that the promotion of BCL-2 transcription by CREB, induced via a YAP-RASAL2 axis, underpins the mechanism of RASAL2-driven chemoresistance in triple-negative breast cancer (TNBC) (Koh et al, 2021; Man et al, 2025).

Furthermore, CREB plays a role in cellular metabolic processes in breast cancer. For instance, it enhances breast cancer cell survival and promotes brain metastasis by regulating glucose transporter protein 3–mediated glucose metabolism (Kuo et al, 2019). Additionally, CREB can directly mediate the expression of vasodilator-stimulated phosphoprotein, a key oncogene in breast cancer that plays a crucial role as a cytoskeleton-associated protein in cell migration and tumor metastasis (Hu et al, 2019). Elevated cytokines (eg, transforming growth factor-*β*, IL-1, and IGF) in the TME have been shown to upregulate CREB, subsequently increasing the expression of the MMP family members (eg, MMP-2, MMP-9, and MMP-13). These MMPs promote extracellular matrix degradation and thereby enable cancer cell invasion, ultimately enhancing the metastatic potential of breast cancer (Kim et al, 2007; Park et al, 2010; Kim et al, 2021b).

Breast cancer gene 1 (*BRCA1*) is a key tumor suppressor gene responsible for DNA damage repair to maintain genomic stability, and 1 of the mechanisms by which it is downregulated is the methylation of CpG dinucleotides in the promoter region (Rice et al, 1998). Mutations or defects in *BRCA1* have long been implicated in the initiation of several cancer types such as breast and ovarian cancers (Romagnolo et al, 2015). Several findings have revealed molecular connections between CREB and *BRCA1*. For example, using supershift gel mobility assay, 1 study shows that the transcription factor that engages with the *BRCA1* promoter consists of a homodimer or heterodimer formed by CREB and activating transcription factor 1 in mammary MCF-7 and T-47D cells (Atlas et al, 2001). Another study using a similar experimental approach shows that aberrant CpG methylation occurs at the binding site of CREB on the *BRCA1* promoter of mammary cells (Mancini et al, 1998). Notably, the interaction of CREB and the promoter region of *BRCA1* is critical for *BRCA1* expression, and any perturbation on the CREB signaling will directly or indirectly affect *BRCA1* expression (Atlas et al, 2001; Ghosh et al, 2008; Shan et al, 2013). Further investigation on the crosstalk between CREB and *BRCA1* is needed to elucidate the functional role of CREB in mediating *BRCA1*-dependent DNA damage response.

The contribution of CREB to the TME, especially in relation to macrophages, has also been noted. One study shows that docetaxel, a commonly used chemotherapeutic drug for breast cancer, can trigger the CCL3-CCR5-p38 pathway by downregulating CREB via reactive oxygen species accumulation in both breast tumor cells and macrophages, ultimately mediating tumor cell phagocytosis by macrophages (Sheng et al, 2022). In another study using highly invasive TNBC cell line MDA-MB-231, activation of the cAMP-PKA-CREB pathway in tumor cells induces an M2 macrophage phenotype in the breast cancer TME to impede the antitumor effects of macrophages (Jiang et al, 2022). Moreover, macrophage-secreted transforming growth factor-*β*1 can promote epithelial-mesenchymal transition and cell migration through activation of the reactive oxygen species–ATM–CREB pathway in breast cancer cell line MCF-7 (Singh et al, 2014). Together, these findings underscore the functional role of CREB in the interaction between tumor cells and macrophages.

Finally, CREB has been found to promote breast cancer bone metastasis through its regulation of parathyroid hormone–related peptide expression (Son et al, 2010). In one study, TNBC cells stably overexpressing CREB were injected into mice, and the implication of CREB in breast cancer-induced osteolytic changes was determined by computed tomography reconstruction and histopathology (Son et al, 2010). The authors found that CREB-induced upregulation of parathyroid hormone–related peptide resulted in trabecular bone loss, facilitating bone metastasis. In addition to metastatic burden, a recent study revealed that inhibiting CREB can sensitize breast cancer cells to chemotherapy (Stevens et al, 2023). In this report, the authors found that elevated cAMP signaling underpins chemoresistance in inflammatory breast cancer, a rare, hard-to-treat, and aggressive subtype of breast cancer. Given that CREB is activated by cAMP, they nominated CREB as one of the candidate therapeutic targets, demonstrating synergistic tumor cell inhibition when CREB inhibitor was combined with chemotherapies doxorubicin and paclitaxel. In another paper, inhibition of multidrug resistance 1 expression has been shown to reverse doxorubicin resistance in the drug-resistant breast cancer cell line MCF-7/adr through activation of the AMPK-GSK-3*β*-CREB pathway (Tran et al, 2013).

In line with the protumor activity of CREB, its expression has been found to correlate with poor prognosis and high histologic grades in patients with breast cancer. In a clinical study involving 153 patients, axillary lymph node-positive patients exhibited significantly higher CREB expression levels compared with axillary lymph node-negative patients (*P* = .0018). Furthermore, disease-free survival was notably reduced in patients with high CREB expression (95.3 [95% CI, 68.4–122.3] months) in contrast to those with low CREB expression [133.9 (95% CI, 123.5–144.2) months, *P* = .0193] (Chhabra et al, 2007). Another prospective study, involving 96 patients, confirmed the inverse correlation between CREB overexpression and good prognosis (Xin et al, 2020). However, further validation with larger patient cohorts is warranted. In particular, it is currently unknown whether CREB predicts survival similarly across all subtypes of breast cancer (ie, hormone receptor-positive, HER2-amplified, and TNBC). Aromatase, encoded by the *CYP19A1* gene, is a key enzyme in estrogen biosynthesis (Artigalás et al, 2015). The *CYP19A1* gene contains multiple specific promoters, and the cAMP/PKA/CREB pathway is considered the primary signaling pathway in regulating the CYP19 promoter (Stocco, 2008). Specifically, phosphorylated CREB regulates the aromatase gene by binding to its promoter region, thereby enhancing its transcription. This mechanism has, in fact, been found to promote breast cancer cell resistance to tamoxifen (Phuong et al, 2014). Thus, harking back to our data implicating CREB in TNBC chemoresistance (Koh et al, 2021; Man et al, 2025), it is conceivable that CREB is also involved in mediating therapeutic resistance in hormone receptor-positive breast cancer.

## 5 CREB as a therapeutic target

**Fig. 3:**
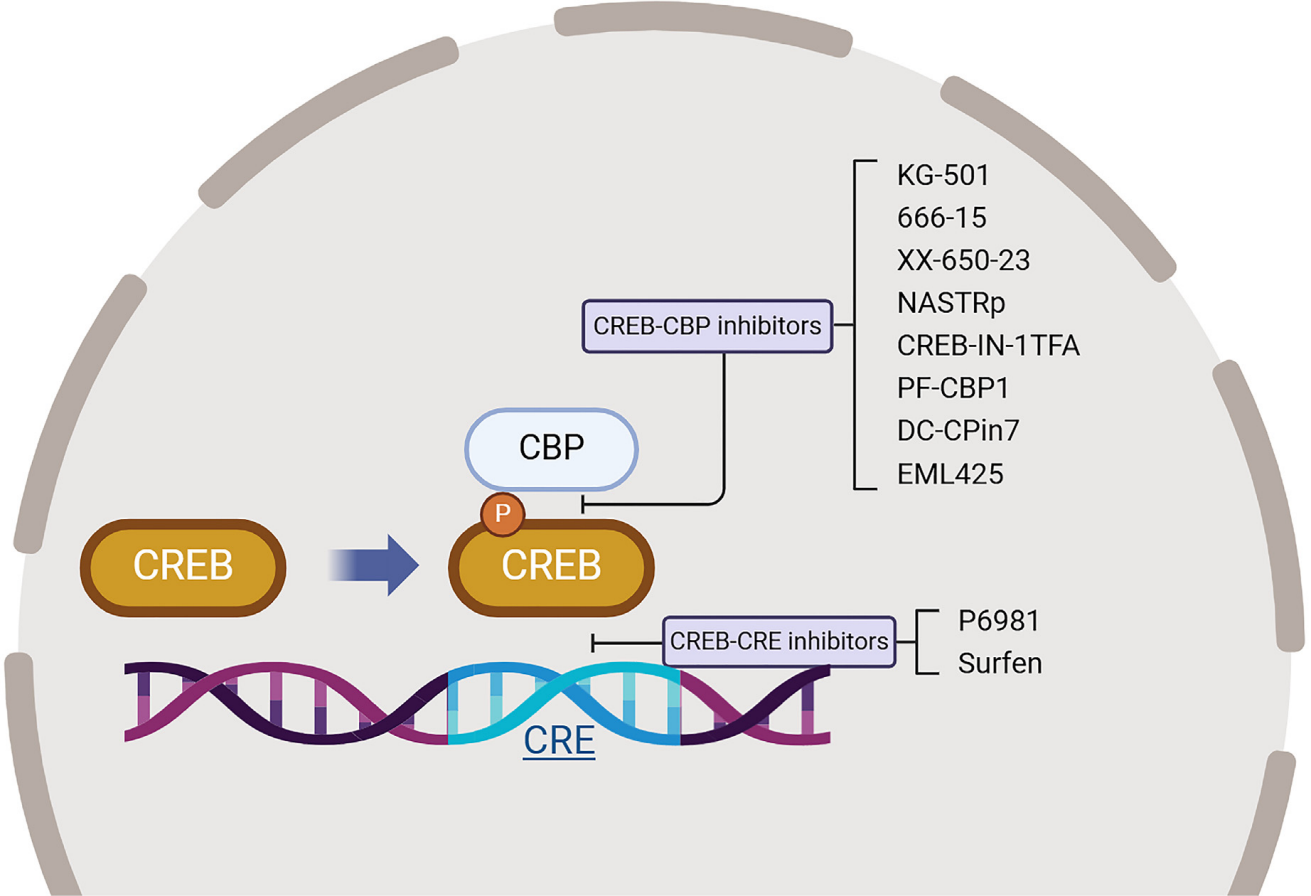
Mechanisms of action of CREB-specific inhibitors. CREB inhibition can be attained by blocking the CREB-CBP interaction or the CREB-CRE interaction.

Given its oncogenic properties, targeting CREB or key nodes along the CREB pathway is a promising yet relatively underexplored avenue. There is ongoing development of CREB-specific inhibitors, which primarily aim at intercepting CREB-DNA and CREB-CBP interactions (Fig. 3). This inhibition can be achieved via 2 strategies: inhibition of the CREB-CRE sequence binding and inhibition of the phosphorylated CREB-CBP binding (Xiao et al, 2010). Although small molecule inhibitors in the first category, such as P6981 and Surfen, have not been extensively studied preclinically in the cancer context (Xiao et al, 2010; Steven et al, 2020), there has been significant preclinical interest in inhibitors of CREB-CBP interaction across various tumor types (Table 1). Among these inhibitors, 666-15 has shown prominent antitumor effects in various TNBC cell lines and patient-derived xenograft models (Xie et al, 2015; Qin et al, 2020). A recent study targeting CREB in a TNBC patient-derived xenograft model found that 666-15 was effective in inhibiting tumor growth when used alone and was even more so when combined with docetaxel (Qin et al, 2020). Treated mice did not experience significant changes in body weight and had no observable toxicities during experimental period, suggesting that the drug has acceptable therapeutic window. Subsequent chemical modification of 666-15 (CREB-IN-1 TFA) led to the development of an oral formulation with a bioavailability of up to 38% (Peng et al, 2022). A summary of preclinical studies on CREB inhibition is provided in Table 2.

**Table 1:** CREB inhibitors and associated preclinical studies

	Name	Molecular Weight	Experimental Model Tested	References
1	KG-501 (NASEp)	377.72	BC (MCF-7, MDA-MB-231) ALL (JURKAT, Molt4, Nalm6, RS4;11) LC (A549, H1734) SS (HS-SY-II, FUJI, 1273/99, CME-1, SYO-I) ProC (LNCaP)	(Steven et al, 2013; Jiang et al, 2019) (van der Sligte et al, 2015) (Sun et al, 2008) (Cyra et al, 2022) (Wang et al, 2016a)
2	666-15	620.52	BC (MCF-7, MDA-MB-231, MDA-MB-468) BC: Human xenograft (MDA-MB-468) PDX (TNBC, PDAC) PDAC (MDA-PATC-148, MiaPaCa2) PC (LNCaP, LAPC-4) LC (H69, H82, H209, H524, A549, H526, H211, H2009) LC: Murine allograft OS (143B) OS: Human xenograft (143B) SS (HS-SY-II, FUJI, 1273/99, CME-1, SYO-I) SS: Human xenograft (SYO-I)	(Xie et al, 2015) (Xie et al, 2015) (Qin et al, 2020; Kim et al, 2021a) (Srinivasan et al, 2018; Kim et al, 2021a) (Pan et al, 2021) (Kim et al, 2022) (Kim et al, 2022) (Li et al, 2021) (Li et al, 2021) (Cyra et al, 2022) (Cyra et al, 2022)
3	XX-650-23	288.3	AML (KG-1, HL-60, MOLM-13, MV-4-11) AML (primary patient cells)	(Mitton et al, 2016) (Mitton et al, 2016)
4	NASTRp	435.71	LC (A549, H441, H1975, H520, H1703) SS (HS-SY-II, FUJI, 1273/99, CME-1, SYO-I)	(Park et al, 2015) (Cyra et al, 2022)
5	CREB-IN-1 TFA	778.06	BC (MDA-MB-231, MDA-MB-468) C57BL/6 mice for pharmacokinetics study	(Peng et al, 2022) (Peng et al, 2022)
6	PF-CBP1	488.62	Untested preclinically, as of 2024	(Chekler et al, 2015)
7	DC-CPin7	358.39	AML (MV-4-11)	(Chen et al, 2020)
8	EML425	440.49	Lymphoma (U-937)	(Milite et al, 2015)
9	P6981	375.95	CCS (SU-CCS-1)	(Zhao et al, 2012)
10	Surfen	372.42	HER-2/neu NIH3T3	(Steven et al, 2017)

ALL, acute lymphoblastic leukemia; AML, acute myeloid leukemia; CCS, clear cell sarcoma; LC, lung cancer; NASE, naphthol-AS-E phosphate; NASTRp, naphthol AS-TR phosphate; OS, osteosarcoma; PDAC, pancreatic ductal adenocarcinoma; ProC, prostate cancer; SS, synovial sarcoma.

**Table 2:** In vivo and in vitro studies involving CREB inhibition in breast cancer

	Year	Model	Mechanism	References
1	2024	BALB/c mice with PyMT-N tumor	Targeting CREB stimulates the immune response by inducing an IFN response and MHC-I expression in breast cancer cells and increasing tumor-infiltrated cytotoxic T cells.	(Yuan et al, 2024)
2	2024	Breast cancer cells (VARI068) and human mammary epithelial cell line (MCF10A)	A dual-targeting ligand called CREBLL-tide (which inhibits the KIX-CREB protein–protein interaction) decreases the cell viability of breast cancer cells (VARI068) but has no effect on the cell viability of nonmalignant mammary epithelial cell line (MCF10A).	(Liu et al, 2024)
3	2023	Breast cancer cells (SUM149 and FCIBC02)	Targeting CREB (CREB inhibitor 666-15) or downregulating CREB decreases breast cancer cells growth and increases sensitivity to paclitaxel.	(Stevens et al, 2023)
4	2022	Breast cancer cells (4T1 and Py8119) and BALB/c mice with 4T1 cells orthotopically injected	Targeting and downregulating CREB can upregulate CCL3, which in turn acts on the Ccr5-p38/Irf5 axis to trigger proinflammatory polarization of macrophages to exert antitumor effects.	(Sheng et al, 2022)
5	2022	Breast cancer cells (MDA-MB-231 and MDA-MB-468)	Targeting CREB (CREB inhibitor 666-15) inhibits breast cancer cell growth.	(Peng et al, 2022)
6	2020	Clinical tissue samples, breast cancer cells (MDA-MB-231, MCF-7, AU565, and T47D), and human mammary epithelial cell line (MCF10A),	CREB expression was significantly higher in patient cancer tissues compared with normal tissues and correlated with disease-free survival and overall survival. CREB expression in all cancer cell lines is significantly higher than in MCF-7 and nonmalignant MCF10A.	(Xin et al, 2020)
7	2020	PDX (TNBC)	Targeting CREB (CREB inhibitor 666-15) inhibits the growth of breast cancer and works even better in combination with docetaxel.	(Qin et al, 2020)
8	2019	Breast cancer cells (MDA-MB-231) and BALB/c mice with MDA-MB-231 cells via tibiae plateau injected	Targeting CREB inhibits osteoclast-specific genes expression and c-Fos/NFATc1 signaling pathway, reversing breast cancer-induced bone loss and breast cancer bone metastasis.	(Jiang et al, 2019)
9	2019	Breast cancer brain metastatic cells (MDA-MB-231 BR and BT474BR)	Knocking down CREB in tumor cells decreases the expression of the glucose transporter protein GLUT3, which is a promoter of breast-to-brain metastasis.	(Kuo et al, 2019)
10	2016	BALB/c mice with MDA-MB-231 cells injected via tail-vein route	Knocking down CREB reduces hypoxia-induced endoplasmic reticulum stress and thus inhibits lung metastasis.	(Kikuchi et al, 2016)
11	2015	Breast cancer cells (MDA-MB-231, A549, MDA-MB-468, and MCF-7) and BALB/c nude mice with MDA-MB-468 cells	Targeting CREB (CREB inhibitor 666-15) inhibits breast cancer cell line growth and tumor growth in MDA-MB-468 xenograft model.	(Xie et al, 2015)
12	2013	Breast cancer cells (MCF-7)	Silencing of CREB downregulates the expression of MMP-2 and MMP-9, thereby affecting the migration and adhesion of breast cancer cells and increasing their susceptibility to apoptosis.	(Steven et al, 2013)

A third CREB-targeting strategy involves the use of inhibitors known to target CREB-related pathways. This approach does not directly target CREB; rather, the goal is to suppress CREB activity. Notably, lapatinib, a common therapeutic drug for breast cancer, has been nominated as a putative CREB-related pathway inhibitor. For example, one report shows that blocking the tyrosine kinase activity of HER-2 using lapatinib downregulates the in vitro and in vivo expression of phosphorylated CREB, which may underpin the antiproliferative and proapoptotic mechanisms of the drug (Steven et al, 2013). For these putative CREB-related pathway inhibitors, further investigation is warranted to elucidate the extent to which the perturbation of the CREB axis contributes to anti-tumorigenic effects (Gschwantler-Kaulich et al, 2016). To date, all studies using CREB inhibitors are at the preclinical stage. However, some of the putative CREB-related pathway inhibitors have approved indications and are being used in the clinic. In addition to lapatinib, these inhibitors include CaMKII inhibitors (eg, ruxolitinib in heart disease) (Reyes Gaido et al, 2023), MAPK inhibitors (eg, trametinib in melanoma) (Thota et al, 2015), PKA inhibitors (eg, sebetralstat in hereditary angioedema) (Riedl et al, 2024), and PI3K inhibitors (eg, idelalisib in non-Hodgkin lymphoma) (Banerjee et al, 2023).

Most studies targeting CREB, either directly or indirectly, have shown tumor inhibitory effect. Although in vivo studies of CREB inhibition have been conducted, predominantly in mouse models (Table 2), further characterization and validation of the safety profile of this approach is warranted. Indeed, CREB is widely distributed in many other cell types including immune cells (Chowdhury et al, 2023), in which CREB plays key roles as described earlier. Therefore, an appropriate threshold of CREB inhibition must be achieved to mitigate untoward off-target effects. To this end, one strategy will be to identify minimal biologically active doses of CREB inhibitors in combination with other drugs, to enable rational drug schedules that maximize tumor-selective killing while minimizing host toxicity (Koh et al, 2018; Koh, 2022). Equally, the development of targeted drug delivery (eg, antibody–drug conjugate) may also reduce systemic toxicity by homing drugs specifically to the tumor site (Perez-Herrero and Fernandez-Medarde, 2015; Jain, 2020). Coupled with in-depth mechanistic understanding, further drug design and development of CREB-targeting agents should yield insights into the feasibility and effectiveness of this approach.

## 6 Conclusions

The treatment landscape of breast cancer has been revolutionized in the past 2 decades by the advent of more targeted options such as poly (ADP-ribose) polymerase inhibitors, PD1/PDL1 axis inhibitors, and antibody–drug conjugates. Given the role of CREB in the development and progression of breast cancer, it is conceivable that targeting this protein represents another viable therapeutic strategy for a subset of hard-to-treat tumors. It is noteworthy that none of the CREB-specific drugs have progressed into clinical trials to date, although several putative CREB-related pathway inhibitors are already in clinical use. One of the outstanding challenges is to identify subsets of patients with breast cancer who would most likely benefit from CREB-targeting treatment. Developing predictive biomarkers of response will be key in enabling patient stratification and ensuring the greatest potential for treatment success. Additionally, to fully rationalize and optimize the application of CREB inhibitors in cancer therapy, future research should focus on: (1) determining the extent CREB inhibition underlies antitumor effect and (2) identifying effective drug combinations and schedules involving CREB-targeting drugs.

## Conflict of interest

The authors declare no conflicts of interest.

## References

[bib1] Ahmed M.B., Alghamdi A.A.A., Islam S.U., Lee J.S., Lee Y.S. (2022). cAMP signaling in cancer: a PKA-CREB and EPAC-centric approach. Cells.

[bib2] Ahn S., Ginty D.D., Linden D.J. (1999). A late phase of cerebellar long-term depression requires activation of CaMKIV and CREB. Neuron.

[bib3] Altarejos J.Y., Montminy M. (2011). CREB and the CRTC co-activators: sensors for hormonal and metabolic signals. Nat Rev Mol Cell Biol.

[bib4] Artigalás O., Vanni T., Hutz M.H., Ashton-Prolla P., Schwartz I.V. (2015). Influence of CYP19A1 polymorphisms on the treatment of breast cancer with aromatase inhibitors: a systematic review and meta-analysis. BMC Med.

[bib5] Asahara H., Santoso B., Guzman E., Du K., Cole P.A., Davidson I., Montminy M. (2001). Chromatin-dependent cooperativity between constitutive and inducible activation domains in CREB. Mol Cell Biol.

[bib6] Atlas E., Stramwasser M., Mueller C.R. (2001). A CREB site in the BRCA1 proximal promoter acts as a constitutive transcriptional element. Oncogene.

[bib7] Bal G., Schneikert J., Li Z., Franke K., Tripathi S.R., Zuberbier T., Babina M. (2023). CREB is indispensable to KIT function in human skin mast cells-a positive feedback loop between CREB and KIT orchestrates skin mast cell fate. Cells.

[bib8] Banerjee T., Kim M.S., Haslam A., Prasad V. (2023). Clinical trials portfolio and regulatory history of idelalisib in indolent non-Hodgkin lymphoma: a systematic review and meta-analysis. JAMA Intern Med.

[bib9] Barco A., Marie H. (2011). Genetic approaches to investigate the role of CREB in neuronal plasticity and memory. Mol Neurobiol.

[bib10] Barzaman K., Karami J., Zarei Z., Hosseinzadeh A., Kazemi M.H., Moradi-Kalbolandi S., Safari E., Farahmand L. (2020). Breast cancer: biology, biomarkers, and treatments. Int Immunopharmacol.

[bib11] Belgacem Y.H., Borodinsky L.N. (2017). CREB at the crossroads of activity-dependent regulation of nervous system development and function. Adv Exp Med Biol.

[bib12] Boulon S., Dantonel J.C., Binet V., Vié A., Blanchard J.M., Hipskind R.A., Philips A. (2002). Oct-1 potentiates CREB-driven cyclin D1 promoter activation via a phospho-CREB- and CREB binding protein-independent mechanism. Mol Cell Biol.

[bib13] Bray F., Laversanne M., Sung H., Ferlay J., Siegel R.L., Soerjomataram I., Jemal A. (2024). Global cancer statistics 2022: GLOBOCAN estimates of incidence and mortality worldwide for 36 cancers in 185 countries. CA Cancer J Clin.

[bib14] Brindle P., Linke S., Montminy M. (1993). Protein-kinase-A-dependent activator in transcription factor CREB reveals new role for CREM repressors. Nature.

[bib15] Chekler E.L., Pellegrino J.A., Lanz T.A., Denny R.A., Flick A.C., Coe J., Langille J., Basak A., Liu S., Stock I.A. (2015). Transcriptional profiling of a selective CREB binding protein bromodomain inhibitor highlights therapeutic opportunities. Chem Biol.

[bib16] Chelakkot V.S., Thomas K., Romigh T., Fong A., Li L., Ronen S., Chen S., Funchain P., Ni Y., Arbesman J. (2023). MC1R signaling through the cAMP-CREB/ATF-1 and ERK-NF*κ*B pathways accelerates G1/S transition promoting breast cancer progression. NPJ Precis Oncol.

[bib17] Chen Y., Bi X., Zhang F., Sun Z., Xu P., Jiang H., Lu W., Lu T., Ding H., Zhang N. (2020). Design, synthesis, and biological evaluation of tetrahydroquinolin derivatives as potent inhibitors of CBP bromodomain. Bioorg Chem.

[bib18] Chhabra A., Fernando H., Watkins G., Mansel R.E., Jiang W.G. (2007). Expression of transcription factor CREB1 in human breast cancer and its correlation with prognosis. Oncol Rep.

[bib19] Chowdhury M.A.R., An J., Jeong S. (2023). The pleiotropic face of CREB family transcription factors. Mol Cells.

[bib20] Chrivia J.C., Kwok R.P., Lamb N., Hagiwara M., Montminy M.R., Goodman R.H. (1993). Phosphorylated CREB binds specifically to the nuclear protein CBP. Nature.

[bib21] Curigliano G., Burstein H.J., Gnant M., Loibl S., Cameron D., Regan M.M., Denkert C., Poortmans P., Weber W.P., Thürlimann B., St Gallen Consensus Conference Panelists 2023 (2023). Understanding breast cancer complexity to improve patient outcomes: the St Gallen International Consensus Conference for the Primary Therapy of Individuals with Early Breast Cancer 2023. Ann Oncol.

[bib22] Cyra M., Schulte M., Berthold R., Heinst L., Jansen E.P., Grünewald I., Elges S., Larsson O., Schliemann C., Steinestel K. (2022). SS18-SSX drives CREB activation in synovial sarcoma. Cell Oncol (Dordr).

[bib23] De Cesare D., Sassone-Corsi P. (2000). Transcriptional regulation by cyclic AMP-responsive factors. Prog Nucleic Acid Res Mol Biol.

[bib24] Dentin R., Hedrick S., Xie J., Yates J., Montminy M. (2008). Hepatic glucose sensing via the CREB coactivator CRTC2. Science.

[bib25] Desdouets C., Matesic G., Molina C.A., Foulkes N.S., Sassone-Corsi P., Brechot C., Sobczak-Thepot J. (1995). Cell cycle regulation of cyclin A gene expression by the cyclic AMP-responsive transcription factors CREB and CREM. Mol Cell Biol.

[bib26] Dinevska M., Widodo S.S., Cook L., Stylli S.S., Ramsay R.G., Mantamadiotis T. (2024). CREB: a multifaceted transcriptional regulator of neural and immune function in CNS tumors. Brain Behav Immun.

[bib27] Ding Y., Huang Y., Zhang F., Gong L., Liang C., Ding K., He X., Ding X., Chen Y. (2023). LncRNA TDRKH-AS1 promotes breast cancer progression via the miR-134-5p/CREB1 axis. J Transl Med.

[bib28] Esvald E.E., Tuvikene J., Sirp A., Patil S., Bramham C.R., Timmusk T. (2020). CREB family transcription factors are major mediators of BDNF transcriptional autoregulation in cortical neurons. J Neurosci.

[bib29] Fan C.F., Mao X.Y., Wang E.H. (2012). Elevated p-CREB-2 (ser 245) expression is potentially associated with carcinogenesis and development of breast carcinoma. Mol Med Rep.

[bib30] Ghosh S., Lu Y., Hu Y. (2008). A role of CREB in BRCA1 constitutive promoter activity and aromatase basal expression. Int J Biomed Sci.

[bib31] Gonzalez G.A., Montminy M.R. (1989). Cyclic AMP stimulates somatostatin gene transcription by phosphorylation of CREB at serine 133. Cell.

[bib32] Gschwantler-Kaulich D., Grunt T.W., Muhr D., Wagner R., Kölbl H., Singer C.F. (2016). HER specific TKIs exert their antineoplastic effects on breast cancer cell lines through the involvement of STAT5 and JNK. PLoS One.

[bib33] Hai T., Curran T. (1991). Cross-family dimerization of transcription factors Fos/Jun and ATF/CREB alters DNA binding specificity. Proc Natl Acad Sci U S A.

[bib34] Han H.S., Kwon Y., Koo S.H. (2020). Role of CRTC2 in metabolic homeostasis: key regulator of whole-body energy metabolism?. Diabetes Metab J.

[bib35] Herzig S., Hedrick S., Morantte I., Koo S.H., Galimi F., Montminy M. (2003). CREB controls hepatic lipid metabolism through nuclear hormone receptor PPAR-gamma. Nature.

[bib36] Herzig S., Long F., Jhala U.S., Hedrick S., Quinn R., Bauer A., Rudolph D., Schutz G., Yoon C., Puigserver P. (2001). CREB regulates hepatic gluconeogenesis through the coactivator PGC-1. Nature.

[bib37] Hoeffler J.P., Meyer T.E., Yun Y., Jameson J.L., Habener J.F. (1988). Cyclic AMP-responsive DNA-binding protein: structure based on a cloned placental cDNA. Science.

[bib38] Hu P., He J., Liu S., Wang M., Pan B., Zhang W. (2016). Beta2-adrenergic receptor activation promotes the proliferation of A549 lung cancer cells via the ERK1/2/CREB pathway. Oncol Rep.

[bib39] Hu P.C., Li K., Tian Y.H., Pan W.T., Wang Y., Xu X.L., He Y.Q., Gao Y., Wei L., Zhang J.W. (2019). CREB1/Lin28/miR-638/VASP interactive network drives the development of breast cancer. Int J Biol Sci.

[bib40] Jain K.K. (2020). An overview of drug delivery systems. Methods Mol Biol.

[bib41] Jiang H., Wei H., Wang H., Wang Z., Li J., Ou Y., Xiao X., Wang W., Chang A., Sun W. (2022). Zeb1-induced metabolic reprogramming of glycolysis is essential for macrophage polarization in breast cancer. Cell Death Dis.

[bib42] Jiang M., Yan Y., Yang K., Liu Z., Qi J., Zhou H., Qian N., Zhou Q., Wang T., Xu X. (2019). Small molecule nAS-E targeting cAMP response element binding protein (CREB) and CREB-binding protein interaction inhibits breast cancer bone metastasis. J Cell Mol Med.

[bib43] Johannessen M., Delghandi M.P., Moens U. (2004). What turns CREB on?. Cell Signal.

[bib44] Johannessen M., Delghandi M.P., Seternes O.M., Johansen B., Moens U. (2004). Synergistic activation of CREB-mediated transcription by forskolin and phorbol ester requires PKC and depends on the glutamine-rich Q2 transactivation domain. Cell Signal.

[bib45] Kang J., Chun J., Hwang J.S., Pan C., Li J., Boese A.C., Young I., Malin C.M., Kang Y., Gibbons D.L. (2022). EGFR-phosphorylated GDH1 harmonizes with RSK2 to drive CREB activation and tumor metastasis in EGFR-activated lung cancer. Cell Rep.

[bib46] Karpinski B.A., Morle G.D., Huggenvik J., Uhler M.D., Leiden J.M. (1992). Molecular cloning of human CREB-2: an ATF/CREB transcription factor that can negatively regulate transcription from the cAMP response element. Proc Natl Acad Sci U S A.

[bib47] Kikuchi D., Tanimoto K., Nakayama K. (2016). CREB is activated by ER stress and modulates the unfolded protein response by regulating the expression of IRE1alpha and PERK. Biochem Biophys Res Commun.

[bib48] Kilanowska A., Ziółkowska A., Stasiak P., Gibas-Dorna M. (2022). cAMP-dependent signaling and ovarian cancer. Cells.

[bib49] Kim E.S., Sohn Y.W., Moon A. (2007). TGF-beta-induced transcriptional activation of MMP-2 is mediated by activating transcription factor (ATF)2 in human breast epithelial cells. Cancer Lett.

[bib50] Kim K.B., Kabra A., Kim D.W., Xue Y., Huang Y., Hou P.C., Zhou Y., Miranda L.J., Park J.I., Shi X. (2022). KIX domain determines a selective tumor-promoting role for EP300 and its vulnerability in small cell lung cancer. Sci Adv.

[bib51] Kim M.P., Li X., Deng J., Zhang Y., Dai B., Allton K.L., Hughes T.G., Siangco C., Augustine J.J., Kang Y. (2021). Oncogenic KRAS recruits an expansive transcriptional network through mutant p53 to drive pancreatic cancer metastasis. Cancer Discov.

[bib52] Kim N.H., Sung N.J., Shin S., Ryu D.S., Youn H.S., Park S.A. (2021). Sauchinone inhibits the proliferation, migration and invasion of breast cancer cells by suppressing Akt-CREB-MMP13 signaling pathway. Biosci Rep.

[bib53] Koh S.B. (2022). The expanding role of WEE1. Cell Signal.

[bib54] Koh S.B., Ross K., Isakoff S.J., Melkonjan N., He L., Matissek K.J., Schultz A., Mayer E.L., Traina T.A., Carey L.A. (2021). RASAL2 confers collateral MEK/EGFR dependency in chemoresistant triple-negative breast cancer. Clin Cancer Res.

[bib55] Koh S.B., Wallez Y., Dunlop C.R., Bernaldo de Quiros Fernandez S., Bapiro T.E., Richards F.M., Jodrell D.I. (2018). Mechanistic distinctions between CHK1 and WEE1 inhibition guide the scheduling of triple therapy with gemcitabine. Cancer Res.

[bib56] Kuo M.H., Chang W.W., Yeh B.W., Chu Y.S., Lee Y.C., Lee H.T. (2019). Glucose transporter 3 is essential for the survival of breast cancer cells in the brain. Cells.

[bib57] Lalli E., Sassone-Corsi P. (1994). Signal transduction and gene regulation: the nuclear response to cAMP. J Biol Chem.

[bib58] Li G., Jiang Q., Xu K. (2019). CREB family: a significant role in liver fibrosis. Biochimie.

[bib59] Li H., Shen X., Ma M., Liu W., Yang W., Wang P., Cai Z., Mi R., Lu Y., Zhuang J. (2021). ZIP10 drives osteosarcoma proliferation and chemoresistance through ITGA10-mediated activation of the PI3K/AKT pathway. J Exp Clin Cancer Res.

[bib60] Li Y., Liu Z., Zhao Y., Yang J., Xiao T.S., Conlon R.A., Wang Z. (2023). PD-L1 expression is regulated by ATP-binding of the ERBB3 pseudokinase domain. Genes Dis.

[bib61] Li Y., Zhang H., Merkher Y., Chen L., Liu N., Leonov S., Chen Y. (2022). Recent advances in therapeutic strategies for triple-negative breast cancer. J Hematol Oncol.

[bib62] Liang H., Tang L.Y., Ge H.Y., Chen M.M., Lu S.Y., Zhang H.X., Shen C.L., Shen Y., Fei J., Wang Z.G. (2023). Neuronal survival factor TAFA2 suppresses apoptosis through binding to ADGRL1 and activating cAMP/PKA/CREB/BCL2 signaling pathway. Life Sci.

[bib63] Liu Y., Joy S.T., Henley M.J., Croskey A., Yates J.A., Merajver S.D., Mapp A.K. (2024). Inhibition of CREB binding and function with a dual-targeting ligand. Biochemistry.

[bib64] Lonze B.E., Ginty D.D. (2002). Function and regulation of CREB family transcription factors in the nervous system. Neuron.

[bib65] Loriaux M.M., Brennan R.G., Goodman R.H. (1994). Modulatory function of CREB.CREM alpha heterodimers depends upon CREM alpha phosphorylation. J Biol Chem.

[bib66] Mallappa S., Neeli P.K., Karnewar S., Kotamraju S. (2019). Doxorubicin induces prostate cancer drug resistance by upregulation of ABCG4 through GSH depletion and CREB activation: relevance of statins in chemosensitization. Mol Carcinog.

[bib67] Man K.-F., Darweesh O., Hong J., Thompson A., O’Connor C., Bonaldo C., Melkonyan M.N., Sun M., Patel R., Ellisen L.W. (2025). CREB1-BCL2 drives mitochondrial resilience in RAS GAP-dependent breast cancer chemoresistance. Oncogene.

[bib68] Mancini D.N., Rodenhiser D.I., Ainsworth P.J., O'Malley F.P., Singh S.M., Xing W., Archer T.K. (1998). CpG methylation within the 5ʹ regulatory region of the BRCA1 gene is tumor specific and includes a putative CREB binding site. Oncogene.

[bib69] Martin L.J., Nguyen H.T. (2022). Basic leucine zipper transcription factors as important regulators of Leydig cells’ functions. Int J Mol Sci.

[bib70] Martinez-Yamout M.A., Nasir I., Shnitkind S., Ellis J.P., Berlow R.B., Kroon G., Deniz A.A., Dyson H.J., Wright P.E. (2023). Glutamine-rich regions of the disordered CREB transactivation domain mediate dynamic intra- and intermolecular interactions. Proc Natl Acad Sci U S A.

[bib71] Mayr B., Montminy M. (2001). Transcriptional regulation by the phosphorylation-dependent factor CREB. Nat Rev Mol Cell Biol.

[bib72] Milite C., Feoli A., Sasaki K., La Pietra V., Balzano A.L., Marinelli L., Mai A., Novellino E., Castellano S., Tosco A. (2015). A novel cell-permeable, selective, and noncompetitive inhibitor of KAT3 histone acetyltransferases from a combined molecular pruning/classical isosterism approach. J Med Chem.

[bib73] Mitton B., Chae H.D., Hsu K., Dutta R., Aldana-Masangkay G., Ferrari R., Davis K., Tiu B.C., Kaul A., Lacayo N. (2016). Small molecule inhibition of cAMP response element binding protein in human acute myeloid leukemia cells. Leukemia.

[bib74] More L., Privitera L., Perrett P., Cooper D.D., Bonnello M.V.G., Arthur J.S.C., Frenguelli B.G. (2022). CREB serine 133 is necessary for spatial cognitive flexibility and long-term potentiation. Neuropharmacology.

[bib75] Nyormoi O., Bar-Eli M. (2003). Transcriptional regulation of metastasis-related genes in human melanoma. Clin Exp Metastasis.

[bib76] Pan W., Zhang Z., Kimball H., Qu F., Berlind K., Stopsack K.H., Lee G.M., Choueiri T.K., Kantoff P.W. (2021). Abiraterone acetate induces CREB1 phosphorylation and enhances the function of the CBP-p300 complex, leading to resistance in prostate cancer cells. Clin Cancer Res.

[bib77] Park J.K., Park S.H., So K., Bae I.H., Yoo Y.D., Um H.D. (2010). ICAM-3 enhances the migratory and invasive potential of human non-small cell lung cancer cells by inducing MMP-2 and MMP-9 via Akt and CREB. Int J Oncol.

[bib78] Park J.M., Greten F.R., Wong A., Westrick R.J., Arthur J.S., Otsu K., Hoffmann A., Montminy M., Karin M. (2005). Signaling pathways and genes that inhibit pathogen-induced macrophage apoptosis--CREB and NF-kappaB as key regulators. Immunity.

[bib79] Park S.A., Platt J., Lee J.W., López-Giráldez F., Herbst R.S., Koo J.S. (2015). E2F8 as a novel therapeutic target for lung cancer. J Natl Cancer Inst.

[bib80] Parry G.C., Mackman N. (1997). Role of cyclic AMP response element-binding protein in cyclic AMP inhibition of NF-kappaB-mediated transcription. J Immunol.

[bib81] Peng J., Miller M., Li B.X., Xiao X. (2022). Design, synthesis and biological evaluation of prodrugs of 666-15 as inhibitors of CREB-mediated gene transcription. ACS Med Chem Lett.

[bib82] Perez-Herrero E., Fernandez-Medarde A. (2015). Advanced targeted therapies in cancer: drug nanocarriers, the future of chemotherapy. Eur J Pharm Biopharm.

[bib83] Persengiev S.P., Green M.R. (2003). The role of ATF/CREB family members in cell growth, survival and apoptosis. Apoptosis.

[bib84] Phuong N.T., Lim S.C., Kim Y.M., Kang K.W. (2014). Aromatase induction in tamoxifen-resistant breast cancer: role of phosphoinositide 3-kinase-dependent CREB activation. Cancer Lett.

[bib85] Qi L., Saberi M., Zmuda E., Wang Y., Altarejos J., Zhang X., Dentin R., Hedrick S., Bandyopadhyay G., Hai T. (2009). Adipocyte CREB promotes insulin resistance in obesity. Cell Metab.

[bib86] Qin Y., Chen W., Jiang G., Zhou L., Yang X., Li H., He X., Wang H.L., Zhou Y.B., Huang S. (2020). Interfering MSN-NONO complex-activated CREB signaling serves as a therapeutic strategy for triple-negative breast cancer. Sci Adv.

[bib87] Revilla Y., Granja A.G. (2009). Viral mechanisms involved in the transcriptional CBP/p300 regulation of inflammatory and immune responses. Crit Rev Immunol.

[bib88] Reyes Gaido O.E., Pavlaki N., Granger J.M., Mesubi O.O., Liu B., Lin B.L., Long A., Walker D., Mayourian J., Schole K.L. (2023). An improved reporter identifies ruxolitinib as a potent and cardioprotective CaMKII inhibitor. Sci Transl Med.

[bib89] Rice J.C., Massey-Brown K.S., Futscher B.W. (1998). Aberrant methylation of the BRCA1 CpG island promoter is associated with decreased BRCA1 mRNA in sporadic breast cancer cells. Oncogene.

[bib90] Riedl M.A., Farkas H., Aygören-Pürsün E., Psarros F., Soteres D.F., Staevska M., Cancian M., Hagin D., Honda D., Melamed I. (2024). Oral sebetralstat for on-demand treatment of hereditary angioedema attacks. N Engl J Med.

[bib91] Romagnolo A.P., Romagnolo D.F., Selmin O.I. (2015). BRCA1 as target for breast cancer prevention and therapy. Anticancer Agents Med Chem.

[bib92] Sakamoto K., Karelina K., Obrietan K. (2011). CREB: a multifaceted regulator of neuronal plasticity and protection. J Neurochem.

[bib93] Sapio L., Salzillo A., Ragone A., Illiano M., Spina A., Naviglio S. (2020). Targeting CREB in cancer therapy: a key candidate or one of many? An update. Cancers (Basel).

[bib94] Saura C.A., Cardinaux J.R. (2017). Emerging roles of CREB-regulated transcription coactivators in brain physiology and pathology. Trends Neurosci.

[bib95] Shan J., Dsouza S.P., Bakhru S., Al-Azwani E.K., Ascierto M.L., Sastry K.S., Bedri S., Kizhakayil D., Aigha I.I., Malek J. (2013). TNRC9 downregulates BRCA1 expression and promotes breast cancer aggressiveness. Cancer Res.

[bib96] Shankar D.B., Sakamoto K.M. (2004). The role of cyclic-AMP binding protein (CREB) in leukemia cell proliferation and acute leukemias. Leuk Lymphoma.

[bib97] Shankar E., Krishnamurthy S., Paranandi R., Basu A. (2010). PKCepsilon induces Bcl-2 by activating CREB. Int J Oncol.

[bib98] Shaywitz A.J., Greenberg M.E. (1999). CREB: a stimulus-induced transcription factor activated by a diverse array of extracellular signals. Annu Rev Biochem.

[bib99] Sheng D., Ma W., Zhang R., Zhou L., Deng Q., Tu J., Chen W., Zhang F., Gao N., Dong M. (2022). Ccl3 enhances docetaxel chemosensitivity in breast cancer by triggering proinflammatory macrophage polarization. J Immunother Cancer.

[bib100] Shnitkind S., Martinez-Yamout M.A., Dyson H.J., Wright P.E. (2018). Structural basis for graded inhibition of CREB:DNA interactions by multisite phosphorylation. Biochemistry.

[bib101] Singh R., Shankar B.S., Sainis K.B. (2014). TGF-beta1-ROS-ATM-CREB signaling axis in macrophage mediated migration of human breast cancer MCF7 cells. Cell Signal.

[bib102] Son J., Lee J.H., Kim H.N., Ha H., Lee Z.H. (2010). cAMP-response-element-binding protein positively regulates breast cancer metastasis and subsequent bone destruction. Biochem Biophys Res Commun.

[bib103] Srinivasan S., Totiger T., Shi C., Castellanos J., Lamichhane P., Dosch A.R., Messaggio F., Kashikar N., Honnenahally K., Ban Y. (2018). Tobacco carcinogen-induced production of GM-CSF activates CREB to promote pancreatic cancer. Cancer Res.

[bib104] Steven A., Friedrich M., Jank P., Heimer N., Budczies J., Denkert C., Seliger B. (2020). What turns CREB on? And off? And why does it matter?. Cell Mol Life Sci.

[bib105] Steven A., Leisz S., Massa C., Iezzi M., Lattanzio R., Lamolinara A., Bukur J., Müller A., Hiebl B., Holzhausen H.J. (2013). HER-2/neu mediates oncogenic transformation via altered CREB expression and function. Mol Cancer Res.

[bib106] Steven A., Leisz S., Wickenhauser C., Schulz K., Mougiakakos D., Kiessling R., Denkert C., Seliger B. (2017). Linking CREB function with altered metabolism in murine fibroblast-based model cell lines. Oncotarget.

[bib107] Steven A., Seliger B. (2016). Control of CREB expression in tumors: from molecular mechanisms and signal transduction pathways to therapeutic target. Oncotarget.

[bib108] Stevens L.E., Peluffo G., Qiu X., Temko D., Fassl A., Li Z., Trinh A., Seehawer M., Jovanović B., Alečković M. (2023). JAK-STAT signaling in inflammatory breast cancer enables chemotherapy-resistant cell states. Cancer Res.

[bib109] Stocco C. (2008). Aromatase expression in the ovary: hormonal and molecular regulation. Steroids.

[bib110] Sun H., Chung W.C., Ryu S.H., Ju Z., Tran H.T., Kim E., Kurie J.M., Koo J.S. (2008). Cyclic AMP-responsive element binding protein- and nuclear factor-kappaB-regulated CXC chemokine gene expression in lung carcinogenesis. Cancer Prev Res (Phila).

[bib111] Tabrizian N., Nouruzi S., Cui C.J., Kobelev M., Namekawa T., Lodhia I., Talal A., Sivak O., Ganguli D., Zoubeidi A. (2023). ASCL1 is activated downstream of the ROR2/CREB signaling pathway to support lineage plasticity in prostate cancer. Cell Rep.

[bib112] Thota R., Johnson D.B., Sosman J.A. (2015). Trametinib in the treatment of melanoma. Expert Opin Biol Ther.

[bib113] Tran T.P., Kim H.G., Choi J.H., Na M.K., Jeong H.G. (2013). Reversal of P-glycoprotein-mediated multidrug resistance is induced by mollugin in MCF-7/adriamycin cells. Phytomedicine.

[bib114] Tsui K.H., Wu M.Y., Lin L.T., Wen Z.H., Li Y.H., Chu P.Y., Li C.J. (2019). Disruption of mitochondrial homeostasis with artemisinin unravels anti-angiogenesis effects via auto-paracrine mechanisms. Theranostics.

[bib115] van der Sligte N.E., Kampen K.R., ter Elst A., Scherpen F.J., Meeuwsen-de Boer T.G., Guryev V., van Leeuwen F.N., Kornblau S.M., de Bont E.S. (2015). Essential role for cyclic-AMP responsive element binding protein 1 (CREB) in the survival of acute lymphoblastic leukemia. Oncotarget.

[bib116] Wang F., Marshall C.B., Ikura M. (2013). Transcriptional/epigenetic regulator CBP/p300 in tumorigenesis: structural and functional versatility in target recognition. Cell Mol Life Sci.

[bib117] Wang J., Yang Z.H., Chen H., Li H.H., Chen L.Y., Zhu Z., Zou Y., Ding C.C., Yang J., He Z.W. (2016). Nemo-like kinase as a negative regulator of nuclear receptor Nurr1 gene transcription in prostate cancer. BMC Cancer.

[bib118] Wang Y.W., Chen X., Ma R., Gao P. (2016). Understanding the CREB1-miRNA feedback loop in human malignancies. Tumour Biol.

[bib119] Wang Z., Zhang L., Ni Z., Sun J., Gao H., Cheng Z., Xu J., Yin P. (2015). Resveratrol induces AMPK-dependent MDR1 inhibition in colorectal cancer HCT116/L-OHP cells by preventing activation of NF-*κ*B signaling and suppressing cAMP-responsive element transcriptional activity. Tumour Biol.

[bib120] Wen A.Y., Sakamoto K.M., Miller L.S. (2010). The role of the transcription factor CREB in immune function. J Immunol.

[bib121] Wu D., Zhau H.E., Huang W.C., Iqbal S., Habib F.K., Sartor O., Cvitanovic L., Marshall F.F., Xu Z., Chung L.W. (2007). cAMP-responsive element-binding protein regulates vascular endothelial growth factor expression: implication in human prostate cancer bone metastasis. Oncogene.

[bib122] Xiao X., Li B.X., Mitton B., Ikeda A., Sakamoto K.M. (2010). Targeting CREB for cancer therapy: friend or foe. Curr Cancer Drug Targets.

[bib123] Xie F., Li B.X., Kassenbrock A., Xue C., Wang X., Qian D.Z., Sears R.C., Xiao X. (2015). Identification of a potent inhibitor of CREB-mediated gene transcription with efficacious in vivo anticancer activity. J Med Chem.

[bib124] Xin Z.C., Hu H.W., Lao Z.H., Zhu L.Q., Biskup E., Zhang H.W. (2020). p-CREB-1 at Ser 133 is a potential marker for breast cancer. Eur Rev Med Pharmacol Sci.

[bib125] Yan Y., Li X., Kover K., Clements M., Ye P. (2013). CREB participates in the IGF-I-stimulation cyclin D1 transcription. Dev Neurobiol.

[bib126] Yuan X., Hao X., Chan H.L., Zhao N., Pedroza D.A., Liu F., Le K., Smith A.J., Calderon S.J., Lieu N. (2024). CREB-binding protein/P300 bromodomain inhibition reduces neutrophil accumulation and activates antitumor immunity in triple-negative breast cancer. JCI Insight.

[bib127] Yun Y.D., Dumoulin M., Habener J.F. (1990). DNA-binding and dimerization domains of adenosine 3ʹ,5ʹ- cyclic monophosphate-responsive protein CREB reside in the carboxyl-terminal 66 amino acids. Mol Endocrinol.

[bib128] Zhang H., Kong Q., Wang J., Jiang Y., Hua H. (2020). Complex roles of cAMP-PKA-CREB signaling in cancer. Exp Hematol Oncol.

[bib129] Zhang J., Ma Y., Wang S., Chen F., Gu Y. (2015). C/EBPalpha inhibits proliferation of breast cancer cells via a novel pathway of miR-134/CREB. Int J Clin Exp Pathol.

[bib130] Zhang N., Shi L., Wang Y. (2022). CREB-associated glycosylation and function in human disease. Adv Clin Exp Med.

[bib131] Zhang Y., Han X.X., Lin X.M., Li Z., Zhang J.H. (2022). miR-450a exerts oncosuppressive effects in breast carcinoma by targeting CREB1. Kaohsiung J Med Sci.

[bib132] Zhao J., Stagno J.R., Varticovski L., Nimako E., Rishi V., McKinnon K., Akee R., Shoemaker R.H., Ji X., Vinson C. (2012). P6981, an arylstibonic acid, is a novel low nanomolar inhibitor of cAMP response element-binding protein binding to DNA. Mol Pharmacol.

[bib133] Zhou W.J., Wang S., Hu Z., Zhou Z.Y., Song C.J. (2015). *Angelica sinensis* polysaccharides promotes apoptosis in human breast cancer cells via CREB-regulated caspase-3 activation. Biochem Biophys Res Commun.

